# Development of the Training Program on Child Abuse Prevention for Citizens (TCAP-C) and Its Effects and Acceptability: Community-Based Participatory Research

**DOI:** 10.3390/ijerph20021414

**Published:** 2023-01-12

**Authors:** Sachiko Kita, Kayoko Ochiai, Yoichi Sato, Saburo Akiyama, Mitsushiro Abe, Keiichi Tashita, Hiroko Tanaka, Fumiko Matsumoto, Shihoko Hayashi, Kosuke Kohashi, Keiichiro Tsujino, Kentaro Uchiyama, Konomi Tsukamatsu, Utako Ikeda, Mari Ikeda, Hidehiro Suzuki

**Affiliations:** 1 Department of Family Nursing, Division of Health Sciences & Nursing, Graduate School of Medicine, The University of Tokyo, Tokyo 1130033, Japan; 2 Department of Health Quality and Outcome Research, Global Nursing Research Center, Graduate School of Medicine, The University of Tokyo, Tokyo 1130033, Japan; 3 Department of Health Policy, National Center for Child Health and Development, Tokyo 1578535, Japan; 4 Association Momrings, Tokyo 1350003, Japan; 5 Kameido 6-Chome Higashi Town, Koto-City, Tokyo 1360071, Japan; 6 NPO KOTO Parent-Child Center/Home Start KOTO, Tokyo 1360072, Japan; 7 Western Federation of Koto-City Juvenile Organization Liaison Council, Tokyo 1350016, Japan; 8 Koto District Youth Committee, Koto-City, Tokyo 1350016, Japan; 9 Children and Youth Affairs, Koto-City, Tokyo 1350016, Japan; 10 Independent Researcher, Koto-City, Tokyo 1360072, Japan; 11 Kamogawa Kokuho Municipal Hospital, Chiba 2960112, Japan; 12 Department of Pediatric Dentistry, Tokyo Dental College, Tokyo 1010061, Japan; 13 Japan Green Clinic, Orchard Road, Singapore 238859, Singapore; 14 San-Ikukai Hospital, Tokyo 1300012, Japan; 15 Association Positive Discipline Community, Tokyo 4080307, Japan; 16 College of Risk Management, Nihon University, Tokyo 1028275, Japan

**Keywords:** child abuse, citizens, community-based participatory research, intervention, prevention, training

## Abstract

Aim: We developed the Training Program on Child Abuse Prevention for Citizens (TCAP-C) and tested its effects and acceptability among citizen leaders (CLs). Methods: Community-based participatory research using a pretest–posttest follow-up design was conducted in Tokyo, Japan from September 2021 to March 2022. Participants completed questionnaires before, upon completion, and one month and three months after TCAP-C. Recognition, knowledge, and behaviors regarding child abuse and community consciousness were collected and compared before and one and three months after TCAP-C, and the degree of satisfaction, understanding, and meaningfulness were collected upon completion. We analyzed data using repeated-measures ANCOVA. Results: A total of 111, 98, 101, and 94 participants completed the questionnaires before, upon completion, and one and three months after TCAP-C, respectively. Overall, the recognition, knowledge, and community consciousness scores significantly improved from before to one month and three months after TCAP-C. Regarding the behaviors, only the behaviors of learning and watching over were significantly improved from before to one month after TCAP-C; however, those behaviors were not different between before and three months after TCAP-C. Furthermore, 95% participants reported being entirely satisfied with TCAP-C, and 85% and 91% reported good understanding and meaningfulness of the program. Conclusions: TCAP-C is acceptable and can improve CL recognition, knowledge, and community consciousness.

## 1. Introduction

Child abuse and neglect (CAN) is a common and serious health issue worldwide. Approximately three in four children aged 2–4 years are exposed to physical punishment and/or psychological violence by parents, and one in five girls and one in thirteen boys aged less than 17 years have been sexually abused globally [1]. A population-based study in Canada reported that the rate of neglect for children in the household was from 20.6% to 29.4%, depending on the children’s age [2]. In Japan, one in five infants at three months after childbirth experienced maltreatment [3], and 78 children aged under 18 years old died due to CAN by parents (i.e., persons who have custody of the child) in 2020 [4]. The number of cases of CAN reported to Children and Family Support Centers (CFSCs)/Child Guidance Centers (CGCs), which are front-line institutions to protect abused children and respond to reports of CAN, reached 360,925 in 2020, which is the highest figure yet recorded [4,5]. CAN causes several lifelong adverse physical, mental, and behavioral health consequences owing to the disruption of early brain development accompanying extreme stress among abused children. These include posttraumatic stress disorder (PTSD), depression, anxiety, high-risk sexual behaviors, unintended pregnancy, smoking, obesity, alcohol and drug misuse, perpetrating or being a victim of violence, withdrawal, heart disease, suicide, and eating disorders [1,3,6,7,8]. In addition, CAN incurs enormous economic costs, including costs of hospitalization, mental health treatment, child welfare, and long-term health costs throughout the victim’s lifetime [6]. A Japanese study [9] reported that the social cost of CAN was approximately $16 billion in 2012 which was almost the same as the total cost of damages ($19 billion) caused by the 2011 Tohoku earthquake and tsunami in Fukushima Prefecture. In addition, the Convention of the Rights of the Child in Japan [10] mentions that all children should grow up in appropriate environments with happiness and understanding in order to develop their personalities, and CAN violates their basic rights. Development of a multi-layered system in collaboration with not only multidisciplinary professionals but also all adults, including the general public (e.g., citizens), is urgently needed to prevent, detect, and protect abused children in the community more effectively, promptly, and appropriately.

Citizens may contribute significantly to solving major practical issues in preventing, detecting, and responding to CAN. Children and families with a higher risk of CAN are more likely not to seek help or remain in contact with appropriate facilities, such as CFSCs, CGCs, hospitals, and welfare offices than children and families with a lower risk of CAN because of the fear of being abused or being abusive, inappropriate recognition and knowledge of CAN, lack of information on social resources, and distrust of other people [11,12,13,14]. In addition, it is extremely difficult for children to find the necessary information on social resources and seek help by themselves because of their immature cognitive, social, and intellectual abilities. Such situations may lead to greater isolation of high-risk children and families in the community, which could contribute to worsening CAN and homicide among children. CAN professionals, such as CFSCs, CGCs, and hospitals, generally intervene for children and families seeking help, reporting, or being visited/delivered for CAN. It is extremely difficult to identify potential high-risk children and families in the community and provide continuous support for them in daily life due to a shortage of information, manpower, and time [14]. As there may thus be a large gap between the help needed by children and families at high risk of CAN and CAN professionals, the provision of social resources to link the gap between them should be mandatory. The strength of citizens is that they live in the same community as children and parents in need of support; thus, they are more likely to discover and notice changes in their living and health conditions (e.g., hearing a child’s intense cries from a neighbor’s house), watch over them more closely, and provide more continuous and delicate support that meets their needs in daily life (e.g., advice on childrearing) as familiar neighbors [15]. The strengths of citizens and their networks could bridge the gap between high-risk children/families and professionals. Thus, empowering citizens and strengthening and using their networks are important for developing effective systems and policies to identify and provide sufficient interventions for all children and families at higher risk of CAN.

Citizen leaders (CLs) may play an important role in developing communities without CAN. *Citizen engagement* has been recognized to be important in improving decision making and developing more effective public health policy as well as developing the knowledge and capacity of citizens [16]. Previous studies [17,18] reported that citizen engagement could effectively and efficiently detect and reduce specific health concerns such as loneliness, depression, anxiety, and prehospital trauma, especially in resource-poor settings or periods. *Citizen leadership* has been defined as an activity in which citizens have power, influence, and responsibility to make decisions and also where citizens take action for the benefit of others [19]. In Japan, CLs are defined as citizens who engage in volunteer activities in the community in cooperation with governmental and other professional facilities, such as CFSCs, CGCs, schools, hospitals, and NPOs/NGOs [15]. These include members of the district committee on youth affairs (DCYM), neighborhood associations (NAs), parent-teacher associations (PTAs), and commissioners of children and youth affairs (CCCYs). Their activities aim to activate and strengthen their community and network and enhance healthy development among children in their community through the planning and operation of annual events (e.g., summer festivals) and children’s camps, and attending conferences with professionals such as CFSCs and teachers to discuss concerned children. CLs are active citizens who are closer to professionals and are more familiar with children and their families in the community; thus, CLs could be key to effectively bridging the gaps between high-risk children/families and professionals. However, only 0.1% of CCCYs and 13.0% of neighbors have reported CAN to CFSCs and CGCs [4]. The main reasons for these low numbers may include a lack of appropriate recognition and knowledge of CAN, lower self-efficacy, indifference, and psychological barriers to reporting and responding to CAN as well as Japanese traditional norms regarding the family (e.g., family issues are private and should not be intervened in) [14,20,21]. Although training programs to acquire and improve their recognition, knowledge, and behaviors regarding CAN have been developed and implemented for health and educational professionals in previous studies [22,23,24,25], an effective training program focusing on citizens (CLs: laypersons) has not yet been developed. Social Cognitive Theory [26,27,28] suggests that increasing self-efficacy is essential to change behaviors, and it is influenced by the following four factors: enactive mastery experience, vicarious learning/modeling, verbal persuasion, and physical and psychological arousal. In addition, a study in Japan [29] has demonstrated that a higher sense of self-determination and solidity in the community was associated with citizens’ behaviors of reporting CAN, possibly due to a specific Japanese culture that values harmony with surroundings more than individuals’ actions. These findings indicate that a training program using team-based and interactive learning contents and techniques should be necessary for CLs to increase their self-efficacy and sense of solidity in the community, not only their knowledge, which would contribute to finally changing their actions and behaviors.

This study aimed to develop a training program on child abuse prevention for citizens (TCAP-C) and investigate its effects and acceptability using a community-based participatory approach. This study is a necessary initial step for increasing and spreading appropriate recognition, knowledge, and behaviors among CLs and other citizens to identify and report CAN effectively and efficiently and provide sufficient support for high-risk children and families in the community. It may prevent and reduce the occurrence and aggravation of CAN and enhance the health and development of children in the community on a long-term basis.

## 2. Methods

### 2.1. Study Design

Community-based participatory research was conducted using a pretest–posttest follow-up design. This pretest–posttest follow-up design was used to identify the effects and acceptability of TCAP-C preliminary, due to a high possibility of contamination among the study participants who were living in the same community and knew each other well.

### 2.2. Study Setting and Period

This study was conducted in a city in Tokyo, Japan between September 2021 and March 2022. The city is located in the southeast of Tokyo, and 16.2% of the residents were children under 18 years old, and 21.1% for seniors over 65 years old [30].

### 2.3. Participants

Citizens who participated in any activity in the city were potential participants of this study. Eligible participants were (1) aged 20–80 years and (2) had sufficient Japanese ability. Citizens who were not available to participate in TCAP-C on the date we suggested were excluded from this study.

### 2.4. Development and Contents of TCAP-C

TCAP-C was developed to improve not only participants’ knowledge but also their appropriate recognition of CAN, motivations, and self-efficacy in preventing/detecting/responding to CAN, which would lead to actions and behaviors to prevent/detect/respond to CAN appropriately in the community among CLs through discussions in a multidisciplinary project team consisting of six administrative officers of a Children and Family Support Center (CFSC) in the city, which is a public facility that accepts and responds to reports of CAN; five staff members of nongovernment organizations; three researchers: two nursing researchers and one legal researcher; five clinical professionals: two pediatricians: one certified nurse midwife, one pediatric dentist, and one certified pediatric emergency nurse; and seven CLs and a citizen from the city: one head and one member of the District Committee on Youth Affairs (DCYM): one chief commissioner of children and youth affairs (CCCY); one head of a neighborhood association (NA); and one foster parent for abused children. The study groups were conducted among the multidisciplinary team monthly from September 2021 to June 2022 to acquire sufficient knowledge and recognition among all the team members, especially the CLs, through lectures on topics such as the concept of CAN, how to prevent/detect/respond to CAN, and important points when dealing with abused children (e.g., prevention of secondary victimization) by professionals focusing on CAN, such as the heads of CFSCs and CGCs, clinical professionals (e.g., pediatricians and pediatric dentists), and researchers. Concurrently, careful discussions on organizing and developing the components and detailed contents of the TCAP-C, including the contents of lectures and the scenarios/worksheets used for group work from several perspectives with respect to their expertise and background, were conducted among the team monthly. After completing the development of the provisional version of TCAP-C, a pilot test for five administrative staff of the CFSC and five citizens who were not team members was conducted in July 2021 to confirm the validity and understanding of the contents, feasibility, and acceptability of TCAP-C. After that, TCAP-C was finalized through discussions and revisions were made by the team according to comments from participants in the pilot test.

**Figure 1:**
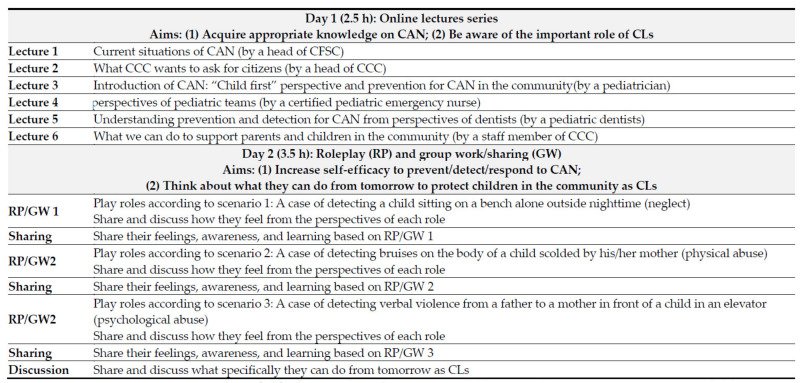
Training program on Child Abuse Prevention for Citizens (TCAP-C). CAN = Child Abuse and Neglect; CFSC = Children Family Support Center; CCC = Children Counseling Center; CL = Citizen leaders.

TCAP-C was a two-day hybrid program (i.e., online and onsite) that lasted for a total of six hours and consisted of two parts: lectures on Day 1 and roleplay and group discussions on Day 2. On Day 1, six lectures were conducted online (each lecture lasted 20–30 min, for a total of 2.5 h) by one head of CFSC, one head and one staff member of CGC, and three clinical professionals (pediatricians, pediatric dentists, and certified pediatric emergency nurses) on the concept, history, and actual situation of CAN; how citizens can prevent/detect/respond to CAN; and the importance of the roles of citizens in reducing or eliminating CAN and protecting and encouraging children’s health and development in the community. Participants were selected from online and on-site lectures. On Day 2, roleplays using three scenarios and discussions based on roleplays in a small group consisting of five to six participants and one facilitator were conducted on-site. Three scenarios were presented to the participants: one case of a child sitting on a bench alone outside at night (neglect), one case of bruises detected on the body of a child scolded by his/her mother (physical abuse), and one case of detecting verbal violence from a father to a mother in front of a child in an elevator (psychological abuse). The actions of the citizens who detected such situations, such as greeting, talking kindly to the child or parent(s), reporting the event to the CFSC, and conversations between the child, parent(s), and citizens, were described in the scenarios. Participants were asked to choose either the role of the child, parent, or citizen and play the role according to the scenario. Participants who did not play any role were asked to watch the roleplay and note how they felt. After the roleplay, the participants were asked to share and discuss how they felt from the perspectives of each role in the group using worksheets. After each roleplay and group discussion ended, the participants or facilitators in each group were asked to summarize and share their feelings, awareness, and learning in front of all participants. After all three roleplays and group discussions were completed, participants were asked to think about and share what they could do starting the next day to protect children and reduce/eliminate CAN in the community from the perspective of CLs. All facilitators were staff members of nongovernmental organizations, CLs, and researchers from among our team members (Figure 1).

### 2.5. Procedures

Eligible participants were recruited through a flyer to participate in this study by one administrative staff member of the CFSC, five staff members of governmental organizations, and five CLs as project team members. If individuals were interested in this study, they were asked to inform the staff of the nongovernmental organization. After that, they were sent a consent form with detailed explanations of the study and were asked to sign the form and return it to the staff of the nongovernmental organization if they agreed to participate. Once their consent was confirmed, they were sent a questionnaire before the intervention (TCAP-C; Time 1: T1) via mail and asked to complete it and send it back within two weeks. In addition, they were informed of the date/place of intervention at the time. On Day 1 of TCAP-C, all lecture series were tape-recorded and live-streamed to participants. On Day 2, participants were asked to come to the site and participate in roleplays/group work/discussions concerning the three scenarios with the facilitators, as mentioned above. As soon as they completed the intervention, participants were asked to complete a brief questionnaire to assess their acceptance and understanding of TCAP-C. One month and three months after TCAP-C (Times 2 and 3: T2 and T3), questionnaires were sent to the participants via mail for them to complete and send back within two weeks. Participants were not provided incentives for this study.

### 2.6. Measures

**Demographic.** Data on age, sex, educational status, having a child, and years and types of activities in the community were collected.

**Recognitions.** Twenty original items were developed based on components of the TCAP-C and discussions in the project team to measure recognition of CAN and motivations/self-efficacy on preventing/detecting/responding to CAN in the community before (T1), one month (T2), and three months (T3) after TCAP-C (see Appendix A). The original scale has five subscales: (1) Willingness to prevent/detect/respond to CAN (eight items; factor 1), such as “I want to listen to feelings of distressed parents” (reverse-scored item); (2) Appropriate recognitions of CAN (five items; factor 2), such as “Witnessing intimate partner violence between parents is not CAN” (reverse-scored item) and “Loudly scolding or hitting a child is sometimes necessary for childrearing” (reverse-scored item); (3) Confidence in detecting/responding to CAN (three items; factor 3), such as “I do not know what to do toward abused children and their parents/guardians” (reverse-scored item); (4) Willingness to engage with unfamiliar family (two items; factor 4), such as “I am not willing to talk to unfamiliar children” (reverse-scored item); (5) Willingness to engage with family who are concerned, such as “I do not want to get involved with parents suspected of child abuse” (two items; factor 5). The factor structure of this scale was confirmed using exploratory factor analysis. Respondents answered on a five-point Likert scale ranging from 1 (*I do not agree at all*) to 5 (*I agree a lot*), where a higher score indicates more appropriate recognition/higher motivation and self-efficacy. The values of Cronbach’s alphas for the total and subscale scores were α = 0.83 (total), α = 0.75 (factor 1), α = 0.68 (factor 2), α = 0.79 (factor 3), α = 0.90 (factor 4), and α = 0.83 (factor 5), respectively.

**Knowledge.** The degree of appropriate knowledge of CAN and preventing/detecting/responding to CAN at T1, T2, and T3 was measured using 20 original items developed based on the contents of the lecture series of the TCAP-C and discussions in the team (see Appendix A). Respondents were asked to answer “yes (correct)” or “no (wrong)” for each item; some examples include: “Most perpetrators of child sexual abuse are strangers”, “Parental consent is required to report CAN”, “Prevention for CAN should focus on only children, not their parents”, and “The most common sources to report CAN in Japan are medical and educational institutions.” Answers were graded by the staff of nongovernmental organizations, and the sum scores of the correct items were calculated; a higher score indicated a higher degree of appropriate knowledge. Cronbach’s alpha was 0.58 for the total score.

**Behaviors.** Twenty-seven original items were used to measure behaviors related to preventing/detecting/responding to CAN in the previous month at T1, T2, and T3 (see Appendix A). These items were developed according to the components of the TCAP-C and discussions with the team. This scale has five subscales: (1) Learning (three items; factor 1), such as “I searched news and articles on child abuse”; (2) Watching over (five items; factor 2), such as “I approached a child that I concerned about” and “I listened to a child about her/his daily life and worries”; (3) Connecting (nine items; factor 3), such as “I consulted with my neighbors about a child that I concerned about”; (4) Staying close (six items; factor 4), such as “I greeted a children/parents in the community”; and (5) Cooperating (four items; factor 5), such as “I discussed with my neighbors about children and parents in the community.” Respondents answered on a five-point Likert scale ranging from 1 (*none*) to 5 (*more than ten times*), with a higher score indicating a higher frequency of appropriate behaviors. Cronbach’s alphas of the total and subscale scores were α = 0.95 (total), α = 0.73 (learning), α = 0.85 (watching over), α = 0.92 (connecting), α = 0.96 (staying close), and α = 0.83 (cooperating), respectively.

**Community consciousness.** Community consciousness was measured using the short version of the Community Consciousness Scale [28]. This scale has 12 items with four subscales (solidarity, self-determination, attachment, and dependency on others), each with three items that participants scored on a five-point Likert scale from 1 (*I do not think so at all*) to 5 (*I think so a lot*), where a higher score indicates a higher degree of community consciousness. The factor structure was confirmed by confirmatory factor analysis [31], and the scores on this scale have been reported to be related to CAN reporting [29]. Cronbach’s alphas of the total and subscale scores were α = 0.81 (total), α = 0.77 (solidarity), α = 0.72 (self-determination), α = 0.54 (attachment), and α = 0.70 (dependency on others).

**Acceptability.** Acceptability of TCAP-C was assessed using four original items: “Overall, were you satisfied with TCAP-C?”, “Were the contents of the lectures on Day 1 easy for you to understand?”, “Were the roleplays and group work on Day 2 meaningful for you?”, and “Did you understand how to prevent/detect/respond to CAN in the community?” The respondents answered on a five-point Likert scale ranging from 1 (*very good*) to 5 (*not good at all*).

### 2.7. Statistical Analyses

First, descriptive statistics of demographics and the main variables (i.e., recognition, knowledge, behaviors, and community consciousness at times 1–3) were calculated. Next, the total and subscale scores of the main variables were compared from T1 to T2, T1 to T3, and T2 to T3 using repeated-measures ANCOVA adjusting confounding factors (i.e., demographic variables), such as age, sex, educational status, having a child or not, and years of activity in the community, to identify the effects of TCAP-C. Regarding acceptability, the distribution of the responses for each item (*n* [%]) was calculated. In addition, the effect sizes (Δ) were calculated to explore the degree of the efficacy of TCAP-C. All analyses were performed using the Statistical Package for Social Science version 20.0 (SPSS. Inc., Chicago, IL, USA). In addition, the minimum sample size in this study calculated using G Power 3.1 software (Heinrich-Heine-Universität Düsseldorf, Düsseldorf, Germany) was 81 (anticipated effect size: 0.34; desired statistical power level: 0.85; α: 0.05; estimated dropout at three months after the intervention: 26%) based on a previous study [24].

### 2.8. Ethical Considerations

The study protocol was approved by the Ethical Committee of the first author’s affiliation and was registered with the Japan Registry of Clinical Trials. All participants provided written informed consent.

To prevent or reduce psychological pressure/coercion among participants to agree with and continue to participate in this study (e.g., attending the TCAP-C and answering questionnaires), because this study used CBPR, especially including CLs in the city, their intention to agree/continue to participate was confirmed only by staff members of nongovernmental organizations who were not directly involved in their activities in the community. In addition, they carefully explained their intentions, and data from the questionnaires were not shared with CLs or administrative staff members on our team to protect their privacy.

## 3. Results

### 3.1. Flow of Participants

**Figure 2:**
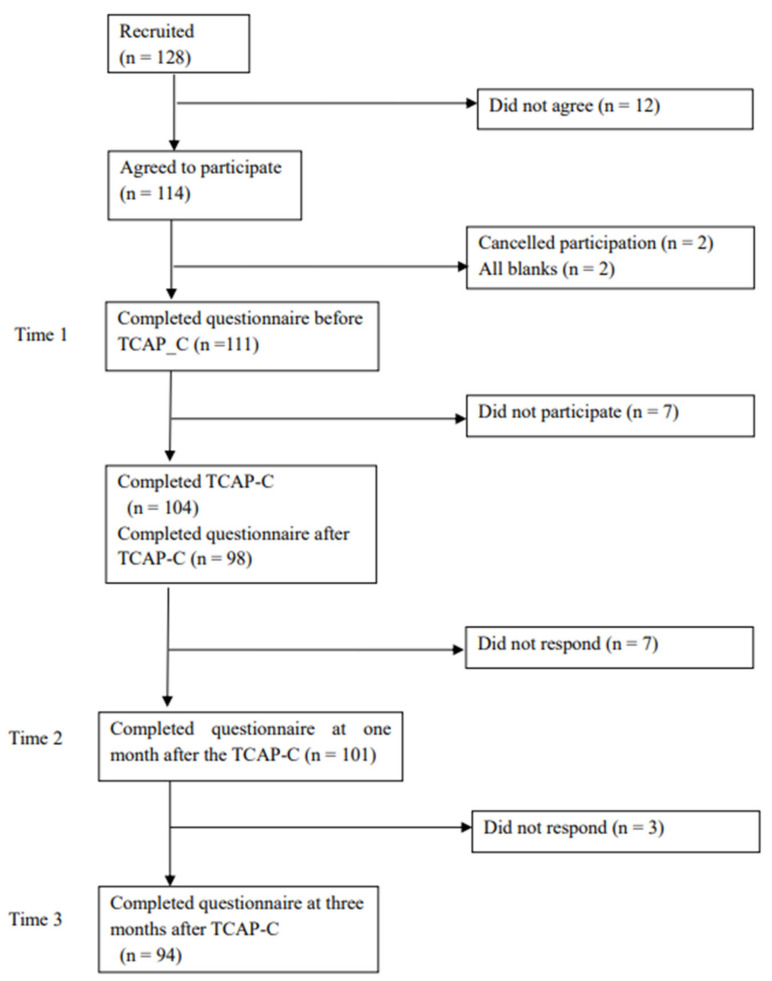
Flow of participants.

A total of 128 participants were recruited, of which 114 agreed to participate in the study. Of these, 111 participants completed the questionnaire before TCAP-C (T1; response rate = 99.1%), and 104 participants completed TCAP-C. Of the 104, 98 participants answered questionnaires asking about the acceptability of TCAP-C as soon as they completed it. At one month (T2) and three months (T3) after TCAP-C, 101 and 94 participants completed the questionnaires (response rates = 97.1% and 90.4%, respectively) (Figure 2).

### 3.2. Participant Demographics

Approximately 84% of the participants were over 50 years old; most were female (73%) and had children (90%). One-third had graduated from senior high school (33%) and 35% were college graduates. The mean number of years of community activity was 13 (0 to 48), and two-thirds were members of a District Committee on Youth Affairs, 34% were members of a neighborhood association, 31% were chief commissioners for children and youth affairs, and 22% were members of a parent–teacher association (Table 1).

### 3.3. Descriptive Statistics at the Baseline

The mean total and subscale scores of recognition before TCAP-C were as follows: total = 77.1, willingness to detect/prevent/respond to CAN = 32.9, appropriate recognition of CAN = 19.5, confidence in detecting/responding to CAN = 9.9, willingness to engage with an unfamiliar family = 7.5, and willingness to engage with the concerned family = 7.4. The mean total knowledge score was 16.4, and the mean total and subscale behavior scores were: 45.3 for total score; 5.5 for learning; 6.5 for watching over, 12.4; 16.2 for staying over; and 5.2 for cooperating. Regarding community consciousness, the total score was 47.8, with scores of 12.0 for solidarity; 11.8 for self-determination; 12.3 for attachment; and 11.6 for dependency on others (Table 2).

### 3.4. Effects of TCAP-C

**Recognition.** The total score and the four subscale scores (i.e., willingness to prevent/detect/respond to CAN, appropriate recognition of CAN; confidence in detecting/responding to CAN; willingness to engage with the concerned family) were significantly improved from before to one month and from before to three months after the TCAP-C. One subscale: willingness to engage with unfamiliar family was not significantly improved from before to one month and three months after TCAP-C (Table 2).

**Knowledge.** The total score (the number of correct answers) significantly increased from before to one month and three months after TCAP-C.

**Behaviors.** The two subscale scores: learning and watching over were significantly improved from before to one month after TCAP-C; however, these were not improved from before to three months after TCAP-C.

**Community consciousness.** The two subscale scores: learning and watching over, were significantly improved from before to one month after TCAP-C; however, these did not improve from before to three months after TCAP-C.

### 3.5. Acceptability of TCAP-C

Approximately 94% of the participants reported being satisfied with the TCAP-C, and 85% and 91% of them answered they were able to understand the contents of the lectures and felt that the roleplays and group discussions were meaningful, respectively. In addition, 95% answered that they were able to understand how to prevent/detect/respond to CAN in the community through the TCAP-C (Table 3).

## 4. Discussion

This study’s results demonstrate that TCAP-C improves overall recognition, knowledge, and community consciousness and partially and temporarily improves CAN behaviors and preventing/detecting/responding to CAN among CLs. In addition, this study showed high acceptability of TCAP-C among CLs.

This study was the first to develop a training program for CLs (laypersons) to prevent/detect/respond to CAN in the community appropriately and to report its good efficacy and acceptability. CLs have a high potential to bridge the gap between high-risk children/families and public facilities, such as CFSCs, CGCs, and hospitals, to protect and enhance the development and health of children in the community [15,16]. However, the majority of CLs have not reported CAN to the facilities [4], possibly due to several barriers including a lack of knowledge and recognition (e.g., self-efficacy), indifference, and psychological hesitation to report CAN [14,20,21]. TCAP-C has been developed using the CBPR approach, respecting citizens’ voices to meet their needs and reduce such barriers. Thus, the contents and techniques used in this program could be practical, especially for CLs, and could successfully reduce their barriers. In addition, the CLs in our team facilitated the roleplays and group discussions, and this might lead to motivating them to learn. In Japan, spreading TCAP-C across the country and training CLs as facilitators to implement this program independently and appropriately should be essential to prevent and terminate CAN in the community in the future. In addition, the approach, contents, and techniques of TCAP-C should be helpful and applicable internationally. Then it could contribute to developing and spreading such training for citizens thus empowering them to protect children’s life, health, and development.

The overall recognition among CLs improved after TCAP-C, as shown by an increase in the total scores and four subscale scores: willingness to prevent/detect/respond to CAN, appropriate recognition of CAN, confidence in detecting/responding to CAN, and willingness to engage with the concerned family. One possible reason is that roleplaying and sharing opinions between the participants could be directly effective in increasing their self-efficacy and motivation to deal with CAN in the community. The determinants of self-efficacy include mastery experiences, vicarious learning, verbal persuasion, and physiological and psychological arousal [26,27,28]. Roleplaying using three different CAN scenarios in more familiar and realistic situations could help CLs to motivate and engage, learn skills used in real-world situations, and imagine what they can do as CLs efficiently. It would result in their successful experience and vicarious leaning, thereby increasing their self-efficacy and motivation to deal with CAN in the community. Indeed, 91% of the participants reported that roleplays/group discussions were meaningful to their learning processes. In contrast, willingness to engage with unfamiliar families did not improve after TCAP-C. This may reflect the influence of Japanese culture, especially in an urban city in Tokyo, including a greater hesitation to engage with unfamiliar people [32]. Thus, the participants’ barriers to unfamiliar families might be difficult to change immediately, even if they completed TCAP-C. Further investigation in different areas, such as rural areas, is necessary to confirm whether the results differ depending on the area.

Furthermore, this study reported improvements in overall knowledge and community consciousness after TCAP-C. Regarding knowledge, the score continuously improved until three months after TCAP-C. Previous studies [33,34] suggest that team-based and interactive learning programs may be more efficient in acquiring and applying knowledge to practice. Those studies indicate that an interactive learning program of TCAP-C could help the participants to acquire and retain their knowledge more effectively. Regarding community consciousness, by participating in TCAP-C and having discussions with other CLs, the participants might feel a rich network between CLs and recognize the importance of the network and their activities to protect and help children and their families in the community, which may lead to increased feelings of attachment and solidarity for their community. A study [16] indicated that mutual trust in citizens is one of the essential factors for citizen engagement in health policy making. In a Japanese study [29], a sense of self-determination and solidity in the community increased participants’ behaviors of reporting CAN. This suggests that because TCAP-C encourages CLs to have mutual communications and exchange their feelings and opinions, it might be helpful in increasing the connection and responsibility to their community. It would lead to empowering CLs to take action to protect children in the community.

Finally, this study reported that TCAP-C did not show an association with improved behaviors three months later, although partial and temporal effects have been reported. Although cognition and acknowledgment could be improved immediately by TCAP-C, behaviors might take longer to change or improve possibly due to higher psychological barriers to actions (e.g., engaging with high-risk families and reporting) such as feelings of fear, hesitation, and guilt [14,20]. Thus, a longer evaluation period to confirm the clear effects of TCAP-C on the behavior and development of a follow-up program after TCAP-C to encourage their actions may be necessary in the future.

### Limitations

This study has some limitations. First, it was conducted using a pre–post design, without a control group. One major reason for this was the higher risk of sharing information on the contents of the intervention between participants if a study with a control group was conducted (i.e., contamination) owing to the features of this study’s participants, such as frequent activity in the community and having close relationships as neighbors and CLs. A future study with a control group conducted in two different cities, such as a cluster randomized controlled trial (RCT), is necessary to reconfirm and clearly identify the effects of TCAP-C.

Second, this study used original scales to measure recognition, knowledge, and behaviors due to a lack of validated scales to measure them, although those original scales have been developed through careful discussion with our team members and showed good reliability using Cronbach’s alphas and one original scale to measure recognition. The primary outcome of this study was confirmed by factor analysis. Further studies to test the validity of these original scales and use the validated scale to confirm the effects of TCAP-C should be conducted in the future.

Third, the demographic characteristics of participants may have influenced this study’s results. This study’s participants were more likely to be older and female because of the features of their living areas (e.g., older town) and activities (e.g., volunteers). Thus, this study’s results should be cautiously interpreted, and a study of participants with more diverse backgrounds may be necessary.

Fourth, this study did not investigate the long-term effects of TCAP-C after three months. In particular, behaviors may take a longer time to improve than recognition and knowledge due to the higher barriers to actions for CAN. A study with a longer evaluation period is needed to test the long-term effects of TCAP-C on the process of the variables after three months, especially behaviors.

Fifth, the CBPR design of this study may have affected participants’ response rates and answers to the questionnaires. The CLs in our project team, who were leaders of the DCYM, CCCY, and NA in the community, were involved in recruitment and intervention in this study. This may have influenced the high response rates of the questionnaires and the desirability bias of their answers, despite careful ethical considerations. Therefore, this study’s results should be cautiously interpreted.

Despite these limitations, this study is the first to develop a training program for CAN for CLs and to confirm its effects and acceptability. This study’s results should be an essential first step in increasing and spreading appropriate recognition, knowledge, and behaviors among citizens to deal with CAN appropriately and effectively in the community, which may contribute to preventing and reducing the occurrence and aggravation of CAN and protecting and enhancing the health, development, and life of children in the community on a long-term basis. To confirm the effects of TCAP-C, a study with a control group (e.g., a cluster RCT) is necessary. In addition, improvement of TCAP-C (e.g., the shortened version and all online programs) may be necessary to increase the feasibility of implementing it for a larger population of CLs and citizens in the future.

## 5. Conclusions

This study was the first to develop a training program regarding CAN for CLs (TCAP-C) and test its effects and acceptability in collaboration with multidisciplinary professionals and citizens in all aspects of the research process. This study’s results demonstrate that TCAP-C significantly improves overall confidence, motivation, knowledge, and community consciousness among CLs, contributing to preventing/detecting/responding to CAN in the community. In addition, this study showed high acceptability of the training program among CLs. This study indicates the importance of spreading such a program to protect children from CAN, which would prevent exposure to violence and enhance life, health, and development among children on a long-term basis.

## Figures and Tables

**Table 1:** Demographics (*n* = 111).

Demographics	*n*/Mean (SD)	(%)/Min–Max
**Age**		
20–29	0	(0.0)
30–39	6	(5.4)
40–49	12	(10.8)
50–59	31	(27.9)
60–69	37	(33.3)
70–79	25	(22.5)
**Sex**		
Female	81	(73.0)
Male	30	(27.0)
Others	0	(0.0)
**Whether or not having a child**		
Yes	100	(90.1)
No	10	(9.0)
Missing	1	(0.9)
**Education**		
Junior high school graduate	4	(3.6)
Senior high school graduate	37	(33.3)
Technical or junior college graduate	29	(26.1)
College graduate	39	(35.1)
Graduate degree	1	(0.9)
Others	1	(0.9)
**Activities in the community** ^a^		
DCYM member ^b^	72	(64.8)
NA member ^c^	38	(34.2)
CCCYA ^d^	34	(30.6)
PTA member ^e^	24	(21.6)
PAC member ^f^	9	(8.1)
FSC member ^g^	8	(7.2)
CSG member ^h^	8	(7.2)
Children’s center staff	7	(6.3)
CSC staff ^i^	6	(5.4)
SSC member ^j^	6	(5.4)
CSG member ^k^	5	(4.5)
Others	25	(22.5)
**Years of the activities**	12.88(10.26)	0–48

^a^ Multiple answers were available; ^b^ DCYM = District Committee on Youth Affairs; ^c^ NA = Neighborhood. association; ^d^ CCCY = Chief commissioner for children and youth affairs; ^e^ PTA = Parent-teacher association; ^f^ PAC = Public assistance committee; ^g^ FSC = Family support cooperative; ^h^ CSG = Childcare support groups: ^i^ CSC= Children’s support center; ^j^ SSC = Short-stay cooperative; ^k^ CSG = Children’s school guidance. Short-stay cooperative.

**Table 2:** Effects of TCAP-C (*n* = 111).

Variables	Time 1 Mean (SD)	Time 2 Mean (SD)	Time 3 Mean (SD)	T1–T2 *p* ^a^ (Δ ^c^)	T1–T3 *p* ^a^ (Δ ^c^)	T2–T3 *p* ^a^ (Δ ^c^)
**Recognitions:** Total score	77.1 (7.8)	80.7 (8.7)	81.1 (8.5)	0.006 (0.46)	0.04 (0.51)	<0.001 (0.05)
Willingness to detect/prevent/respond to CAN	32.9 (3.3)	33.8 (3.7)	33.8 (3.5)	0.008 (0.27)	0.005 (0.27)	0.87 (0.00)
Appropriate recognitions of CAN	19.5 (2.9)	20.5 (2.9)	20.6 (2.8)	<0.001 (0.35)	<0.001 (0.38)	0.14 (0.04)
Confidence in detecting/responding to CAN	9.9 (2.4)	11.3 (2.1)	11.1 (2.1)	<0.001 (5.8)	<0.001 (0.50)	0.31 (−0.01)
Willingness to relate to unfamiliar family	7.5 (1.8)	7.3 (1.9)	7.6 (1.7)	0.25 (−0.11)	0.87 (0.06)	0.12 (1.6)
Willingness to relate to family who are concerned	7.4 (1.4)	7.8 (1.6)	8.0 (1.2)	0.006 (0.29)	<0.001 (0.43)	0.09 (0.13)
**Knowledge:** Total score	16.4 (2.2)	17.4 (1.7)	17.4 (1.8)	<0.001 (0.46)	<0.001 (0.46)	0.23 (0.00)
**Behaviors:** Total score	45.3(18.2)	47.6(18.9)	46.7 (16.3)	0.27 (0.13)	0.82 (0.08)	0.16 (−0.05)
Learning	5.5 (2.3)	6.2 (2.2)	5.9 (2.1)	0.02 (0.31)	0.21 (0.17)	0.26 (−0.14)
Watching over	6.5 (3.2)	7.4 (3.7)	6.9 (3.1)	0.04 (0.28)	0.50 (0.13)	0.03 (−0.14)
Connecting	12.4 (5.9)	12.6 (5.8)	12.3 (4.6)	0.62 (0.03)	0.61 (−0.02)	0.24 (−0.05)
Staying close	16.2 (7.8)	16.7 (7.5)	16.5 (7.6)	0.75 (0.07)	0.92 (0.04)	0.77 (−0.03)
Cooperating	5.2 (3.1)	5.1 (2.7)	5.0 (2.7)	0.59 (−0.03)	0.39 (−0.04)	0.79 (−0.07)
**Community consciousness ^b^:** Total score	47.8 (5.3)	49.1 (5.7)	48.6 (5.8)	0.002 (0.25)	0.005 (0.15)	0.80 (−0.09)
Solidarity	12.0 (2.2)	12.2 (2.7)	12.4 (2.4)	0.45 (0.09)	0.03 (0.18)	0.20 (0.00)
Self-determination	11.8 (1.8)	12.0 (2.1)	12.0 (1.9)	0.14 (0.11)	0.02 (0.11)	0.48 (0.00)
Attachment	12.3 (1.7)	12.7 (1.6)	12.6 (1.7)	0.001 (0.24)	0.02 (0.18)	0.34 (−0.06)
Dependency on others	11.6 (1.8)	12.0 (1.9)	11.7 (1.7)	0.03 (0.22)	0.44 (0.06)	0.20 (−0.16)

Repeated-measure ANCOVA was conducted adjusting demographic variables, such as age, sex, educational status, having a child or not, and years of activity in the community to compare the scores of the three time points. TCAP-C = Training program on Child Abuse Prevention for Citizens; CAN = Child Abuse and Neglect; Time 1/T1 = Before TCAP-C; Time 2/T2 = One month after TCAP-C; Time3/T3 = three months after TCAP-C. ^a^ Calculated using paired-sample *t* test; ^b^ Community consciousness was measured using the short version of the Community Consciousness Scale; ^c^ Effect size.

**Table 3:** Acceptability of TCAP-C (*n* = 98).

	Very Good		Good		Neither		Not Good		Not Good at All	
	*n* (%)		*n* (%)		*n* (%)		*n* (%)		*n* (%)	
Satisfaction of TCAP-C	46	(45.9)	47	(48.0)	5	(5.1)	0	(0.0)	0	(0.0)
Easiness to understand the lectures	31	(31.6)	53	(54.1)	11	(11.2)	0	(0.0)	0	(0.0)
Meaningfulness of the roleplay and group work	38	(38.8)	51	(52.0)	7	(7.1)	1	(1.0)	0	(0.0)
Understanding of how to prevent/detect/respond to CAN	33	(33.7)	61	(62.2)	4	(4.1)	0	(0.0)	0	(0.0)

TCAP-C = Training program on Child Abuse Prevention for Citizens; CAN = Child Abuse and Neglect.

## Data Availability

The data presented in this study are available on request from the corresponding author.

## Appendix A. Questionnaires Regarding Recognition, Knowledge and Behaviors

Recognition: 20 items
1. I believe loudly scolding or hitting a child is sometime necessary for childrearing 2. I feel parenting supports in the community can prevent child abuse 3. I think witness of intimate partner violence is not child abuse 4. I feel it is disrespectful as a citizens to ask about child abuse 5. I think strangers should not get involved in family problems 6. I am uncomfortable talking to children/parents I don’t know 7. I am uncomfortable greeting children/parents I don’t know 8. I am not willing to talk to unfamiliar children 9. I am not willing to talk to unfamiliar parents/guardians 10. I do not want to get involved with parents suspected of child abuse 11. I want to listen to feeling of distressed parents 12. I do not know what to do toward abused children and their parents/guardians 13. I do not know how to detect abused children or their parents/guardians 14. I do not know where to consult with about abused children or their parents/guardians 15. I want to report child abuse to a public institution 16. I feel that reporting child abuse is a gateway to support for abused children and parents 17. I do not want to report child abuse to a public institution 18. I feel that citizens need to be knowledgeable about prevention of child abuse 19. I feel that prevention of child abuse requires a face-to-face relationship between citizens 20. I feel that my community is highly interested in prevention of child abuse
Knowledge: 20 items
1. The number of cases of child abuse reported in this city in 2020 was approximately 700 2. The most common sources to report child abuse in Japan are medical and educational institutions 3. Japan’s response to child abuse is advanced even in the world 4. Child abuse is an act in which a child’s rights are violated 5. Response to child abuse is to find abusers of children 6. Intergenerational transmission of violence cannot be prevented by citizens 7. The purpose of reporting child abuse to a public institution is to provide information and support to children and their families 8. Child abuse is a family problem and there is nothing the community can do 9. Most perpetrators of child sexual abuse are strangers 10. If you find a “concerned parent or child”, you can immediately report to a public institute 11. Evidence of child abuse is necessary for reporting a public institute 12. Consent of parents/guardians is necessary for reporting child abuse to a public institute 13. Dentists play an important role in detecting neglect and physical child abuse 14. Child health checkups are important for early detection of child abuse 15. Early detection of abuse requires involvement in health and dental checkups in the community 16. To protect children from child abuse, we should focus on supporting children 17. Being yelling or hitting by parents develop a sense of “distrust” or “dislike” among the children 18. It is effective for parents to warn or point out each time their children do something wrong 19. The human brain is particularly vulnerable to the influence of the parenting environment during the developmental process from the age of two months to preschool 20. For the healthy development of children’s brains, it is effective for parents and other adults around them to give positive feedbacks to their children.
Behaviors: 27 items
1. I searched news and articles on child abuse 2. I checked out/attended lectures on child abuse 3. I searched child abuse dramas, movies, etc. 4. I observed children in the community 5. I approached a child that I concerned about 6. I asked a concerned child about his/her daily life and worries 7. I approached the parent that I concerned about 8. I asked a concerned parent about his/her life, problems, etc. 9. I talked with my neighbors about child abuse 10. I collected information about children in the community 11. I collected information about child abuse 12. I was consulted by my neighbors who had detected child abuse 13. I was consulted by parents concerning about abuse or parenting 14. I consulted with my neighbors about a child that I concerned about 15. I consulted with a public institute about parents that I concerned about 16. I consulted with my neighbors about a parent that I concerned about 17. I consulted with a public institute about parents that I concerned about 18. I greeted a child in the community 19. I smiled at a child in the community 20. I spoke kindly to a child in the community 21. I greeted parents in the community 22. I smiled at parents in the community 23. I spoke kindly to parents in the community 24. I actively engaged in activities for parents and children in the community 25. I cooperated with the government on behalf of parents and children in the community 26. I discussed with my neighbors for children and parents in the community 27. I discussed with the staff of the government for parents and children in the community
